# Activation of NOTCH1 or NOTCH3 Signaling Skews Human Airway Basal Cell Differentiation toward a Secretory Pathway

**DOI:** 10.1371/journal.pone.0116507

**Published:** 2015-02-20

**Authors:** Kazunori Gomi, Vanessa Arbelaez, Ronald G. Crystal, Matthew S. Walters

**Affiliations:** Department of Genetic Medicine, Weill Cornell Medical College, New York, New York, United States of America; University of Southern California, UNITED STATES

## Abstract

Airway basal cells (BC) function as stem/progenitor cells capable of differentiating into the luminal ciliated and secretory cells to replenish the airway epithelium during physiological turnover and repair. The objective of this study was to define the role of Notch signaling in regulating human airway BC differentiation into a pseudostratified mucociliated epithelium. Notch inhibition with γ-secretase inhibitors demonstrated Notch activation is essential for BC differentiation into secretory and ciliated cells, but more so for the secretory lineage. Sustained cell autonomous ligand independent Notch activation via lentivirus expression of the intracellular domain of each Notch receptor (NICD1-4) demonstrated that the NOTCH2 and 4 pathways have little effect on BC differentiation into secretory and ciliated cells, while activation of the NOTCH1 or 3 pathways has a major influence, with persistent expression of NICD1 or 3 resulting in a skewing toward secretory cell differentiation with a parallel decrease in ciliated cell differentiation. These observations provide insights into the control of the balance of BC differentiation into the secretory *vs* ciliated cell lineage, a balance that is critical for maintaining the normal function of the airway epithelium in barrier defense against the inhaled environment.

## Introduction

Notch signaling is an evolutionarily conserved signaling pathway involved in a wide variety of cellular processes, including turnover and repair of tissues and organs [1–4]. Mammals express five Notch ligands (delta-like ligand 1, 3, 4, jagged 1, 2) and four Notch receptors (Notch1-4), all localized on plasma membranes [2,4]. The Notch receptors are type I transmembrane receptors with both extracellular and intracellular domains. Upon ligand binding, the receptor is cleaved by a γ-secretase at the intracellular transmembrane region, resulting in release of the Notch intracellular domain (NICD) into the cytoplasm. The cleaved NICD translocates to the nucleus and forms an active transcriptional complex with the DNA binding protein recombination signal binding protein for immunoglobulin J-kappa region (RBPJK) and additional co-activators [5,6]. The resulting complex then binds within the promoters of multiple target genes to regulate their expression. Activation of the Notch pathway via different receptor-ligand interactions can result in a diverse array of downstream responses, allowing the Notch pathway to regulate many cellular processes [7].

Murine studies have demonstrated that during development and in the adult lung, Notch signaling regulates differentiation of the airway epithelium into the secretory, Clara, ciliated and neuroendocrine cell types [8–22]. In contrast, little is known regarding the role of Notch signaling in regulating differentiation of the human airway epithelium, a complex tissue composed of basal cells (BC), ciliated, secretory and columnar/undifferentiated cells [23–25]. In both the human and mouse airways, the BC are the proliferating stem/progenitor population that differentiate into the other specialized epithelial cell types of the airway during normal epithelial turnover and repair [26–35].

Based on the knowledge that the Notch signaling pathway is expressed in the human airway epithelium [36], the present study is focused on assessing which of the 4 Notch receptors play a role in regulating the differentiation of human airway BC into secretory and ciliated cells. The data demonstrate that NOTCH2 and 4 have little influence, but that signaling mediated by the NOTCH1 and 3 pathways plays a central role in regulating the differentiation of BC into secretory and ciliated cells, with sustained activation of these pathways skewing differentiation to the secretory lineage. These observations have implications for developing targets to restore normal airway epithelial structure in human airway disorders characterized by increased secretory cell numbers and mucus production.

## Methods

### Ethics Statement

All individuals were evaluated and samples collected in the Weill Cornell NIH Clinical and Translational Science Center and Department of Genetic Medicine Clinical Research Facility under clinical protocols approved by the Weill Cornell Medical College and New York/Presbyterian Hospital Institutional Review Boards (IRB) according to local and national IRB guidelines. All subjects gave their informed written consent prior to any clinical evaluations or procedures.

### Culture of Primary Human Airway Basal Cells

Nonsmoker primary airway basal cells (BC) were obtained either by isolation using selective culture methods from large airway epithelial samples obtained by bronchoscopy under IRB approved protocols as described previously [33] or purchased commercially (Lonza Catalog CC2540S, Walkersville, MD, USA). The BC phenotype of all cells was confirmed by positive staining for the BC markers KRT5, TP63 and CD151 (>99% positive cells) and negative staining for additional differentiated airway epithelial cell types (secretory, ciliated cell and neuroendocrine) as previously described [33]. Representative images depicting characterization of the primary BC are included in S1 Fig. All cultures were seeded at 3000 cells/cm^2^ into plastic flasks and maintained in BEGM medium (Lonza) supplemented with 1% penicillin/streptomycin (GIBCO-Life Technologies, Grand Island, NY, USA), 0.5% amphotericin B (GIBCO-Life Technologies) and 0.1% gentamicin (Sigma, St Louis, MO, USA) in a 5% CO_2_, 37°C humidified incubator. The medium was replaced every 2 to 3 days and once the cells had reached 80% confluence, the cells were harvested for air-liquid interface (ALI) culture by trypsinization in 0.05% trypsin-ethylenediaminetetraacetic acid (EDTA, GIBCO-Life Technologies) at 37°C, 5 min with the reaction stopped by addition of (4-(2-hydroxyethyl)-1-piperazineethanesulfonic acid buffered saline (HEPES, Lonza) supplemented with 15% fetal bovine serum (FBS, GIBCO-Life Technologies). For primary BC obtained via isolation using selective culture methods from large airway epithelial samples obtained by bronchoscopy each independent culture was passaged one round to obtain sufficient cell material before study on ALI culture, whereas cells obtained commercially were thawed directly into T75 flasks and used on ALI culture with no further expansion once the appropriate density was reached. Each independent experiment was performed with cells derived from independent donors.

### RNA Extraction and cDNA Synthesis

Total RNA was extracted using a modified version of the TRIzol method (Invitrogen), in which RNA is purified directly from the aqueous phase using the Rneasy MinElute RNA purification kit (Qiagen, Valencia, CA, USA). RNA samples were stored in RNA Secure (Ambion, Austin, TX, USA) at -80°C. The concentration was determined using a NanoDrop ND-1000 spectrophotometer (NanoDrop Technologies, Wilmington, DE, USA). Double-stranded cDNA was synthesized from 1 μg of total RNA using TaqMan Reverse Transcription Reagents (Applied Biosystems, Foster City, CA, USA),

### Microarray

Genome wide gene expression of nonsmoker derived primary human airway BC (n = 5) was assessed using the HG-U133 Plus 2.0 array (Affymetrix, Santa Clara, CA, USA) as previously described [33]. Briefly, all HG-U133 Plus 2.0 microarrays were processed according to Affymetrix protocols, hardware and software, processed by the Affymetrix fluidics station 450 and hybridization oven 640 and scanned with an Affymetrix Gene Array Scanner 3000 7G. The captured image data from the HG-U133 Plus 2.0 arrays were processed using the MAS5 algorithm in GeneSpring version 7.3 (Affymetrix Microarray Suite Version 5) and normalized per chip to the median expression value of each sample to generate P calls for filtering. Overall microarray quality was verified by the following criteria: (1) RNA Integrity Number (RIN) >7.0; (2) 3′/5′ ratio for GAPDH <3; and (3) scaling factor <10.0. The expression level of Notch pathway genes was assessed using the following HG-U133 Plus 2.0 array probesets (P call ≥20% of all samples): DLL1 (224215_s_at), DLL3 (222898_s_at), DLL4 (223525_at), JAG1 (209099_x_at), JAG2 (32137_at), NOTCH1 (218902_at), NOTCH2 (212377_s_at), NOTCH3 (203238_s_at), NOTCH4 (205247_at), RBPJ (207785_s_at), HES1 (203394_s_at), HES2 (231928_at), HES4 (227347_x_at), HES5 (239230_at), HES6 (226446_at), HES7 (224548_at), HEY1 (44783_s_at), HEY2 (219743_at), HEYL (226828_s_at) and KRT5 (201820_at). The raw data and values are publically available at the Gene Expression Omnibus (GEO) site (http://www.ncbi.nlm.nih.gov/geo/), GSE53537.

### Air-liquid Interface Culture

To investigate the differentiation of human airway BC, the cells were grown on air-liquid interface (ALI) cultures. The BC were trypsinized and seeded at a density of 3.75 x 10^5^ cells/cm^2^ onto 0.4 μm pore-sized Transwell inserts (Corning Incorporated, Corning, NY, USA) pre-coated with human type IV collagen (Sigma). The initial culture medium consisted of a 1:1 mixture of DMEM (Cellgro, Manassas, VA, USA) and Ham's F-12 Nutrient Mix (GIBCO-Invitrogen, Carlsbad, CA, USA) containing 5% fetal bovine serum, 1% penicillin-streptomycin, 0.1% gentamycin and 0.5% amphotericin B. The following day, the medium was changed to 1:1 DMEM/Ham’s F12 (including antibiotics described above) with 2% Ultroser G serum substitute (BioSerpa S.A., Cergy-Saint-Christophe, France). Once the cells had reached confluence (following 2 days of culturing on the membrane), the media were removed from the upper chamber to expose the apical surface to air and establish the ALI (referred to as ALI Day 0). The cells were then grown at 37°C, 8% CO_2_, and the culture medium was changed every 2 to 3 days. Following 5 days on ALI, the cells were grown at 37°C, 5% CO_2_ until harvested.

### Inhibition of Notch Activity with γ-Secretase Inhibitors

To inhibit activation of Notch signaling during BC differentiation, ALI cultures were treated with media containing the γ-secretase inhibitors, N-[N-(3,5-Difluorophenacetyl)-L-alanyl]-S-phenylglycine t-butyl ester (DAPT; Sigma) or (S,S)-2-[2-(3,5-Difluorophenyl)acetylamino]-N-(5-methyl-6-oxo-6,7-dihydro-5H-dibenzo[b,d]azepin-7-yl) propionamide (DBZ; EMD Chemicals Inc., Gibbstown, NJ, USA). DAPT and DBZ were dissolved in dimethyl sulfoxide (DMSO), and added into media at a final concentration of 5 μM and 0.1 μM, respectively, from ALI day 0. Fresh drug was added with every media change for the length of time indicated. As controls, cells were also cultured in untreated media or media containing DMSO (0.1%).

### Histology

Air-liquid interface day 28 transwell inserts were washed three times with PBS and then fixed directly with 4% paraformaldehyde in PBS for 20 min. Following fixation, the samples were washed three times with PBS and then shipped in 70% ethanol to Histoserv, Inc (Germantown, MD, USA) whereupon the membrane was removed from the transwell insert and subsequently dehydrated through graded alcohols, cleared in xylene and then infiltrated with low melt temperature paraffin and sectioned at 5 μm. Before staining, the samples were deparaffinized in xylene substitute, followed by incubation in a series of graded ethanol to rehydrate. For general histology, sections were stained using standard protocols for Alcian blue/Nuclear Fast Red Kernechtrot reagents (Poly Scientific R&D Corp. Bay Shore, NY, USA).

### Quantification of Differentiation

To quantify the differentiation capacity of primary BC following ALI culture, paraffin-embedded sections were stained with Alcian blue/Nuclear Fast Red Kernechtrot reagents to determine the percentage of ciliated and secretory cells or immunofluorescent staining of cell type specific markers. A minimum of 10 images equally distributed between both ends of each sectioned membrane were acquired. Basal cells were determined by positivity for the marker KRT5, ciliated cells were determined by morphology and secretory cells by Alcian blue staining or positivity for the markers MUC5AC and SCGB1A1, where described. For each sectioned membrane, a minimum of 200 total cells were counted.

### TaqMan PCR

Complementary DNA (cDNA) was synthesized from 1 μg of total RNA using TaqMan Reverse Transcription Reagents (Applied Biosystems). All samples were then analyzed in duplicate and all reactions run on an Applied Biosystems Sequence Detection System 7500 with relative expression levels determined using the dCt method with 18S ribosomal RNA as the endogenous control. Premade TaqMan Gene Expression Assays were obtained from Applied Biosystems: KRT5 (Hs00361185_m1); TP63 (Hs00978343_m1); MUC5AC (Hs01365616_m1); SCGB1A1 (Hs00171092_m1); DNAI1 (Hs00201755_m1); TEKT1 (Hs00364985_m1); RPBJK (Hs01061428_g1), HES1 (Hs00172878_m1); HES2 (Hs01021800_g1); HES4 (Hs00368353_g1); HES5 (Hs01387463_g1); HES6 (Hs00936587_g1); HEY1 (Hs01114113_m1); HEY2 (Hs00232622_m1); and HEYL (Hs00232718_m1).

### Western Analysis

Cells were harvested and lysed in radioimmunoprecipitation lysis (RIPA) buffer (Sigma) containing complete protease inhibitor cocktail (Roche, Mannheim, Germany) and halt phosphatase inhibitor cocktail (Pierce, Rockford, IL, USA). The protein concentration was then quantified using the Bradford Assay and an appropriate volume of 4X NuPAGE LDS sample buffer (Invitrogen) containing 200 mM dithiothreitol (DTT) added to each sample. The cellular lysates were then boiled for 10 min and equal amounts of total protein for each sample analyzed using NuPAGE 4–12% Bis–Tris gradient gels (Invitrogen) and subsequently transferred onto nitrocellulose membranes with a Bio-Rad semidry apparatus before Western analysis, as previously described [37]. The primary antibodies used for Western analysis included: rabbit polyclonal KRT5 (basal cell; 1/5000; PA1-37974; Thermo Scientific, Rockford, IL, USA); mouse monoclonal TP63 (basal cell; 1/3000; sc-8431; Santa Cruz Biotechnology, Inc., Santa Cruz, CA, USA); rabbit polyclonal SCGB1A1 (secretory cell; 1/10000; RD181022220; BioVendor LLC, Candler, NC, USA); rabbit polyclonal DNAI1 (ciliated; 1/2000; HPA021649; Sigma); mouse monoclonal anti-human-GAPDH (1/5000, SC-32233, Santa Cruz Biotechnology); rabbit monoclonal anti-NOTCH1 (1/2000, #4380, Cell Signaling Technology, Danvers, MA, USA); rabbit monoclonal anti-NOTCH2 (1/1000, #4530, Cell Signaling Technology); rabbit monoclonal anti-NOTCH3 (1/1000, #5276, Cell Signaling Technology) and mouse monoclonal anti-NOTCH4 (1/1000, #2423, Cell Signaling Technology).

### Immunofluorescence

Immunofluorescent staining was performed either on paraffin embedded cross-sections or directly by top-staining of the ALI membrane. For analysis of paraffin embedded sections, the samples were first cleaned in xylene and rehydrated with graded ethanol. To unmask the antigens, samples were steamed for 15 min in citrate buffer solution (Thermo Scientific) followed by cooling at 23°C for 20 min then permeabilized with 0.1% triton X-100 in PBS for 10 min followed by blocking with normal for 30 min to reduce background staining. For direct top-staining, the ALI membranes were fixed directly with 4% paraformaldehyde for 20 min and then permeabilized with 0.1% triton X-100 in PBS followed by blocking with normal serum. The samples were then treated and stained with the following primary antibodies: β-tubulin IV (ciliated cell; 5 μg/ml; MU178-UC; Biogenex, Fremont, CA); KRT5 (basal cell; 2 μg/ml; PA1-37974; Thermo Scientific); SCGB1A1 (secretory cell; 5 μg/ml; RD181022220; BioVendor LLC); MUC5AC (secretory cell; 1.4 μg/ml; VP-M657; Vector Laboratories, Burlingame, CA), HEY1 (downstream effector; 20 μg/ml; ab22614; Abcam, Cambridge, MA, USA) and HEYL (downstream effector; 20 μg/ml; H00026508-M03, Abnova, Taipei, Taiwan) overnight at 4°C. Isotype matched IgG (Jackson Immunoresearch Laboratories, West Grove, PA, USA) was the negative control. To visualize the antibody binding, Alexa Fluor 488 Goat Anti-Mouse IgG (A-11029; Invitrogen) and Alexa Fluor 546 Goat Anti-Rabbit IgG (A-11035; Invitrogen) labeled secondary antibodies were used. The cells were counterstained with DAPI to identify cell nuclei and subsequently mounted using ProLong Gold antifade reagent (Invitrogen). Immunofluorescent microscopy was performed using a Zeiss Axioplan body microscope with either a 40 x or 100 x lens. The images were captured with a Zeiss hrM (high resolution monochrome) camera.

### Immunohistochemistry

Immunohistochemical staining was performed on paraffin embedded cross-sections of the ALI membrane. The samples were first cleaned in xylene and rehydrated with graded ethanol. To unmask the antigens, samples were steamed for 15 min in citrate buffer solution (Thermo Scientific) followed by cooling at 23°C for 20 min. Endogenous peroxidase activity was quenched using 0.3% H_2_O_2_ for 30 min, followed by incubation for 30 min with normal serum matched to the secondary antibody to reduce background staining. Samples were incubated overnight at 4°C with the following primary antibodies: rabbit monoclonal anti-NOTCH1 (5.0 μg/ml, ab52627, Abcam); rabbit monoclonal anti-NOTCH2 (0.5 μg/ml, #4530, Cell Signaling Technology); rabbit monoclonal anti-NOTCH3 (0.02 μg/ml, ab178948, Abcam); mouse monoclonal anti-NOTCH4 (0.04 μg/ml, #2423, Cell Signaling Technology); HEY1 (0.5 μg /ml; ab22614; Abcam) and HEYL (5.0 μg/ml; H00026508-M03, Abnova). Isotype-matched IgG (Jackson ImmunoResearch Laboratories Inc.) was used as a negative control. The Vectastain Elite ABC kit and AEC substrate kit (Dako North America Inc., Carpinteria, CA, USA) were used to visualize antibody binding and slides were counterstained with Mayer’s hematoxylin (Polysciences Inc., Warrington, PA) and mounted using faramount mounting medium (Dako North America Inc.). Images were acquired using a Nikon Microphot microscope with a Nikon Plan × N.A. 0.70 40 x objective lens and an Olympus DP70 CCD camera.

### Generation of Notch Intracellular Domain Expressing Lentiviruses

For sustained activation of Notch signaling during BC differentiation on ALI culture, the intracellular domain of the human NOTCH1, 2, 3 and 4 receptors (NICD1, 2, 3 and 4, respectively) were cloned into a lentiviral expression plasmid. Nucleotide sequences encompassing NICD1 (5281–7668), NICD2 (5095–7416), NICD3 (5002–6966) and NICD4 (4417–6012) were PCR amplified using gene specific primers (NICD1 Forward 5’-GTATTCTAGAGCTAGCGAATTCGCCACGATGcggcggcagcatggccagct-3’and Reverse 5’-GATCCGATTTAAATTCGAATTCttacttgaaggcctccggaatgcgggcg-3’; NICD2 Forward 5’-GAATTCCCACCatggcaaaacgaaagcgtaagcatggctc-3’ and Reverse 5’-GGATCCtcacgcataaacctgcatgttgttgtgtgg-3’; NICD3 Forward 5’-GAATTCGCCACCATGaagcgcgagcacagcaccctctggttccctg-3’ and Reverse 5’-GGATCCtcaggccaacacttgcctcttgggggtaac-3’ and NICD4 Forward 5’-GAATTCGCCACGATGcgtcgacgccgagagcatggagct-3’ and Reverse 5’-GGATCCctattttttaccctctcctccttggtttatgggc-3’) and cloned into the multiple cloning site of pCDH-MSCV-MCS-EF1α-GFP (Cat No:CD711B-1, System Biosciences, Mountain View, CA, USA) via EcoRI (NICD1) and EcoRI and BamHI (NICD2-4) restriction sites. The cDNA template of NICD1 and NICD3 were purchased from Addgene (Cat No: 17626 and 26894 respectively, Mountain View, CA, USA) and the cDNA templates of NICD2 and 4 were kindly provided by Dr Warren Pear, University of Pennsylvania, USA. Once NICD1-4 were cloned into pCDH-MSCV-MCS-EF1α-GFP, the resulting plasmids (Lenti-NICD1-4) were sequenced to verify the correct orientation and integrity of each open reading frame. In addition, expression of each NICD was confirmed by transfection of 293A cells and subsequent Western analysis with antibodies specific for each gene. Recombinant replication deficient lentiviruses pseudotyped with the VSVg envelope were generated by transient co-transfection of 293A cells with Lenti-NICD1-4 and the appropriate packaging plasmids. The virus containing media was collected at 24 hr intervals with replacement of fresh media at each time point. At 72 hr post transfection the media was harvested and pooled with previous time points for subsequent virus purification by standard methods. The infectious titer of each virus was determined via GFP positivity following infection of an immortalized airway basal cell line (BCi-NS1.1) with serial dilutions of the virus [37].

### Lentivirus Infection of Primary Human Airway Basal Cells on Air-liquid Interface Culture

Primary BC were infected with recombinant lentiviruses at an equal multiplicity of infection (MOI) that allowed for equal infectivity between samples as determined by GFP positivity. For infection of cells on ALI culture, the virus was added directly to the cell suspension at the time of seeding the cells on Transwell inserts in the standard ALI media supplemented with 2 μg/ml of polybrene to aid virus infection. The following day, the infectious media was removed and the standard ALI protocol continued as described above.

### Statistical Analysis

The Mann-Whitney U test was used for statistical analyses of all experiments. In all analyses, a p value less than 0.05 was deemed significant.

## Results

### Expression of Notch Signaling Pathway Components in Human Airway Basal Cells

**Fig 1:**
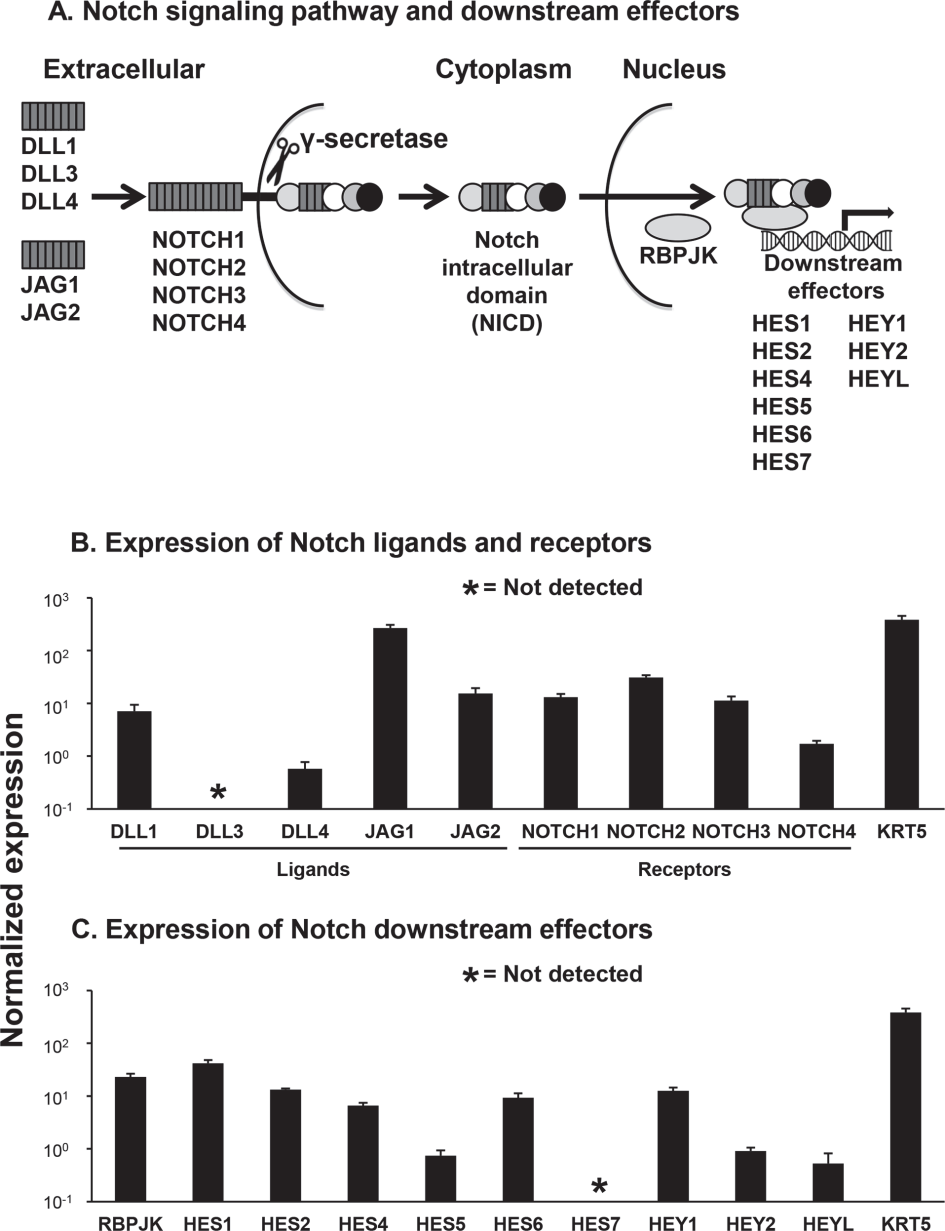
Expression of Notch pathway ligand, receptor and downstream effector genes in human airway basal cells. **A**. Overview of Notch signaling pathway and downstream effectors. Shown are the 5 Notch-ligands [delta-like ligand 1 (DLL1), 3 (DLL3), 4 (DLL4), jagged 1 (JAG1) and 2 (JAG2)], and the 4 Notch receptors (NOTCH1, 2, 3 and 4). The notch intracellular domain (NICD) represents the cytoplasmic portion of each Notch receptor cleaved by the γ-secretase complex. Once translocated to the nucleus, cleaved NICD forms an active transcriptional complex with the DNA binding protein RBPJK. The NICD-RBPJK complex binds Notch target genes and activates Notch downstream effectors, HES1, HES2, HES4, HES5, HES6, HES7, HEY1, HEY2 and HEYL. **B**. Basal cell relative expression of Notch pathway ligands and receptors. **C**. Basal cell relative expression of Notch pathway downstream effectors. For panels **B** and **C**, the HG-U133 Plus 2.0 microarray was used to assess relative expression analysis for Notch pathway ligand, receptor and downstream effector genes. Each bar represents the mean expression level with standard error of the mean for each gene in nonsmoker human airway basal cells (n = 5). The expression level of the basal cell specific marker keratin 5 (KRT5) was used as a reference.

The Notch signaling pathway contains multiple ligands, receptors and downstream effectors (Fig. 1A). Microarray analysis of human airway basal cells (BC) revealed expression of the Notch ligands DLL1, DLL4, JAG1 and JAG2, and expression of the four Notch receptors (NOTCH1-4) (Fig. 1B). However, the ligand DLL3 was not expressed. Analysis of known Notch downstream effectors demonstrated expression of RBPJK, HES1, HES2, HES4, HES5, HES6, HEY1, HEY2 and HEYL, while HES7 was not expressed (Fig. 1C). Overall these data demonstrate that human airway BC express most components of the Notch signaling pathway.

### Notch Activation is Required for Differentiation of Human Airway Basal Cells

**Fig 2:**
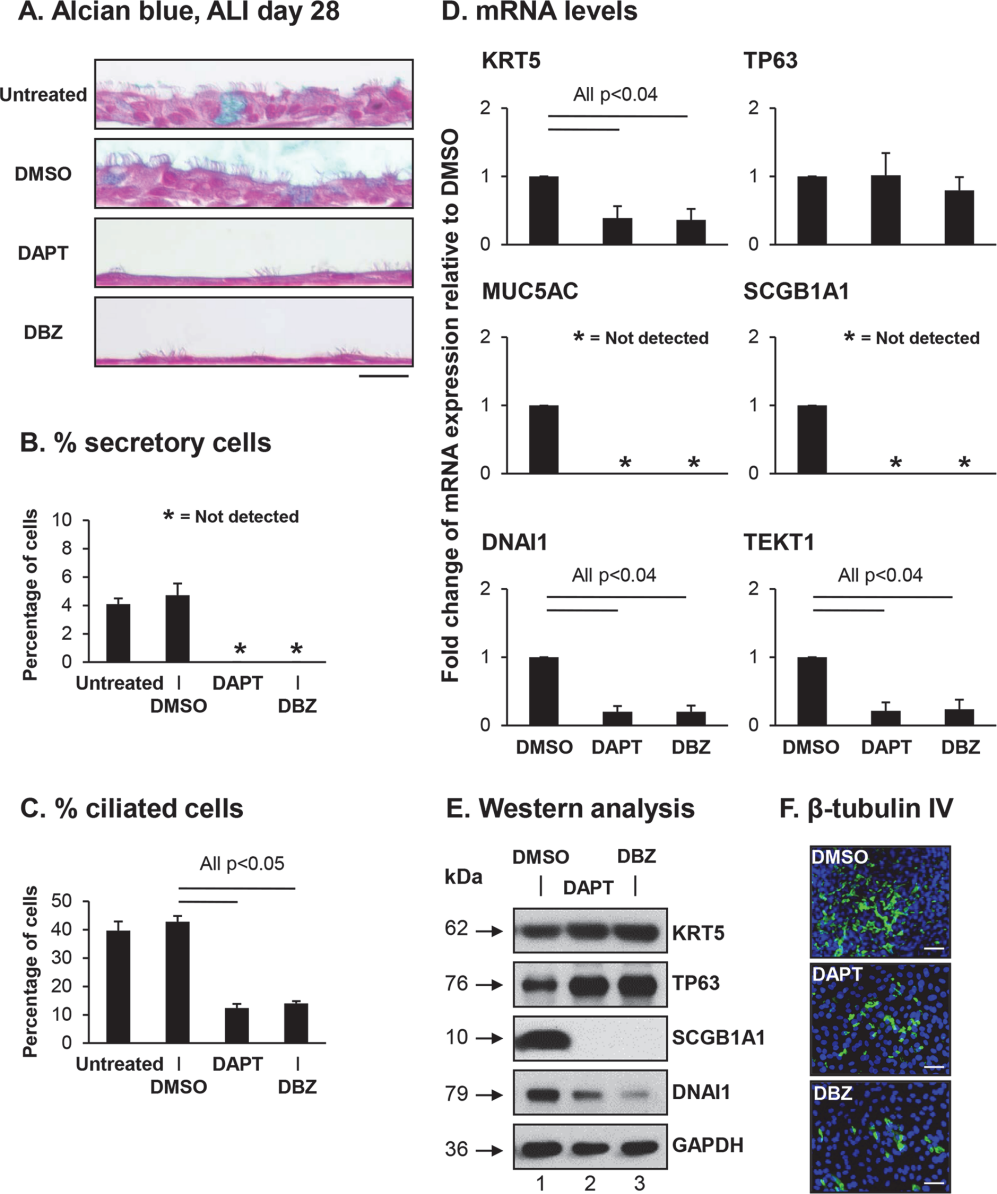
Activation of Notch signaling is required for differentiation of human airway basal cells. Airway basal cells were differentiated on air-liquid interface cultures (ALI) for 28 days in the presence and absence of γ-secretase inhibitors DAPT (5 μM) or DBZ (0.1 μM). Treatment with DMSO (0.1%) was used as the drug solvent control. **A**. Alcian blue staining of ALI day 28 sections. Scale bar 20 μm. **B-C**. Quantification of secretory and ciliated cells on Alcian blue stained ALI day 28 sections. **B**. Percentage secretory cells; and **C**. Percentage ciliated cells. The data for **B** and **C** are the mean for n = 3 independent experiments; error bars indicate standard error of the mean. **D**. mRNA expression for cell type specific markers. TaqMan PCR was used to assess expression of basal cell markers (KRT5 and TP63); secretory cell markers (MUC5AC and SCGB1A1) and ciliated cell markers (DNAI1 and TEKT1). Bars indicate the mean fold-change of mRNA expression compared to DMSO-treated ALI cells from n = 3 independent experiments, each performed in triplicate. Error bars indicate standard error of the mean. **E**. Western analysis for cell type specific markers. Lane 1—DMSO; lane 2—DAPT; and lane 3—DBZ. For all cells, shown is the expression of basal cells markers (KRT5, TP63), secretory cell marker (SCGB1A1) and ciliated cell marker (DNAI1). GAPDH was used as a loading control. Data shown are representative images from n = 3 independent experiments. **F**. Immunofluorescence assessment of the ciliated cell marker β-tubulin IV (green) and DAPI (nuclei, blue). In the panels in descending order, ALI cells cultured in media containing DMSO (0.1%), DAPT (5 μM) or DBZ (0.1 μM). Scale bar 50 μm.

To assess the role of Notch signaling in regulation of human airway BC differentiation, BC were cultured under differentiation-inducing air-liquid interface (ALI) conditions in the presence of γ-secretase inhibitors (DAPT or DBZ) that inhibit activation of Notch signaling. Following 28 days of ALI culture, differentiation of BC into secretory and ciliated cells was quantified at the histological level by Alcian blue staining of ALI day 28 cross-sections (Fig. 2A). As expected, treatment of cells with the drug vehicle DMSO had no significant (both p>0.8) effect on the numbers of secretory (4.1% untreated *vs* 4.7% DMSO) and ciliated cells (39.6% untreated *vs* 42.8% DMSO) relative to untreated cells (Fig. 2B,C). Compared to control DMSO treated cells, continuous treatment of differentiating BC with DAPT or DBZ resulted in generation of a thin single cell layered epithelium with reduced appearance of luminal differentiated cells (Fig. 2A). Quantification of Alcian blue positive cells demonstrated a complete block in differentiation into secretory cells (Fig. 2A, B). Treatment with DAPT and DBZ allowed for differentiation into ciliated cells, although relative to DMSO treated cells, there was a significant decrease in ciliated cell numbers in DAPT (12.4% *vs* 42.8% DMSO, p<0.05) and DBZ (14% *vs* 42.8% DMSO, p<0.05) treated cultures (Fig. 2A, C).

**Fig 3:**
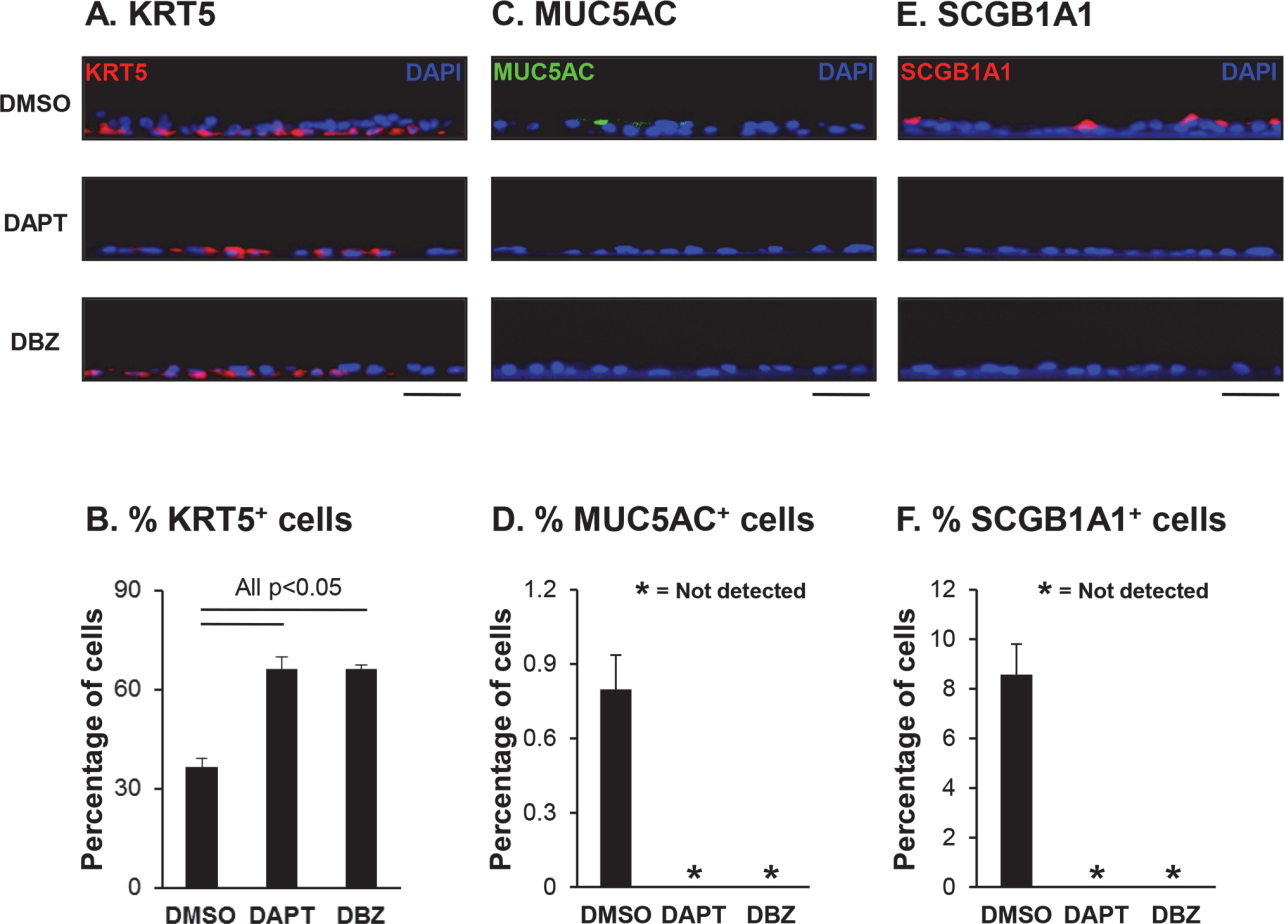
Activation of Notch signaling is required for secretory cell differentiation of human airway basal cells. Airway basal cells were differentiated on air-liquid interface cultures (ALI) for 28 days in the presence and absence of γ-secretase inhibitors DAPT (5 μM) or DBZ (0.1 μM). Treatment with DMSO (0.1%) was used as the drug solvent control. **A**. Immunofluorescence staining of KRT5 positive cells. Sections of cells on ALI day 28 membranes were stained for KRT5 (basal cell, red) and DAPI (nuclei, blue). Scale bar 20 μm. **B**. Quantification of KRT5 positive cells on ALI membranes. **C**. Immunofluorescence staining of MUC5AC positive cells. Sections of cells on ALI day 28 membranes were stained for MUC5AC (secretory cell, green) and DAPI (nuclei, blue). Scale bar 20 μm. **D**. Quantification of MUC5AC positive cells on ALI membranes. **E**. Immunofluorescence staining of SCGB1A1 positive cells. Sections of cells on ALI day 28 membranes were stained for SCGB1A1 (secretory cell, red) and DAPI (nuclei, blue). Scale bar 20 μm. **F**. Quantification of SCGB1A1 positive cells on ALI membranes. The data for **B, D** and **F** are the mean for n = 3 independent experiments; error bars indicate standard error of the mean.

To further characterize the effects of Notch inhibition by DAPT and DBZ treatment on the differentiation capacity of the BC, TaqMan quantitative PCR, Western analysis and immunofluorescent staining using cell type specific markers were performed. TaqMan analysis of ALI day 28 DMSO *vs* DAPT or DBZ treated cells with cell type specific transcript primer-probesets demonstrated significant differences in expression of a subset of cell type specific markers (Fig. 2D). Analysis of the BC marker KRT5 revealed a significant decrease in expression in cells treated with DAPT and DBZ (both p<0.04), but no significant change in expression of the BC marker TP63 (both p>0.4). In support of the histological data, no detectable expression of the secretory cell markers MUC5AC and SCGB1A1 was observed in cells treated with DAPT and DBZ. In addition, there was a significant (both p<0.04) decrease in expression of the ciliated cell markers DNAI1 and TEKT1 in cells treated with DAPT and DBZ relative to DMSO. Surprisingly, Western analysis of the BC markers KRT5 and TP63 contrasted the mRNA data and demonstrated an increase in the levels of protein for KRT5 and TP63 in cells treated with DAPT and DBZ relative to DMSO (Fig. 2E). However, Western analysis of the secretory cell marker SCGB1A1 and ciliated cell marker DNAI1 correlated with the mRNA data with no expression and reduced expression of the respective genes in cells treated with DAPT and DBZ relative to DMSO control (Fig. 2E). Furthermore, immunofluorescent staining of the ciliated cell marker β-tubulin IV showed reduced numbers of ciliated cells in DAPT and DBZ treated cells compared to DMSO controls (Fig. 2F) further confirming the quantification of ciliated cell numbers by morphology (Fig. 2C) and the expression of ciliated cell markers by TaqMan quantitative PCR (Fig. 2D) and Western analysis (Fig. 2E). Differentiation was further validated at the protein level by immunofluorescent staining and quantification of cell numbers for KRT5 (basal cell), MUC5AC (secretory cell) and SCGB1A1 (secretory cell). Relative to DMSO treated cells, for both DAPT and DBZ there were significantly (all p<0.05) higher numbers of KRT5 positive cells (36.3% DMSO vs 66.2% DAPT and 66.1% DBZ) thus confirming the Western data (Fig. 3A-B). Therefore, it appears that Notch inhibition with γ-secretase inhibitors results in a decrease in the expression of BC markers (e.g. KRT5) at the mRNA level, however there is an inverse relationship at the protein level resulting in increased levels through an unknown mechanism. Quantification of MUC5AC and SCGB1A1 positive cells demonstrated the presence of both cell types in DMSO treated cells (0.8%, MUC5AC and 8.5%, SCGB1A1), whereas there was a complete absence of these cell types in cells treated with DAPT and DBZ further confirming the mRNA and Western data (Fig. 3C-F). Overall, these data suggest that activation of Notch signaling is essential for BC differentiation into secretory cells, and required for optimal differentiation into ciliated cells.

### Sustained Activation of Notch Signaling

**Fig 4:**
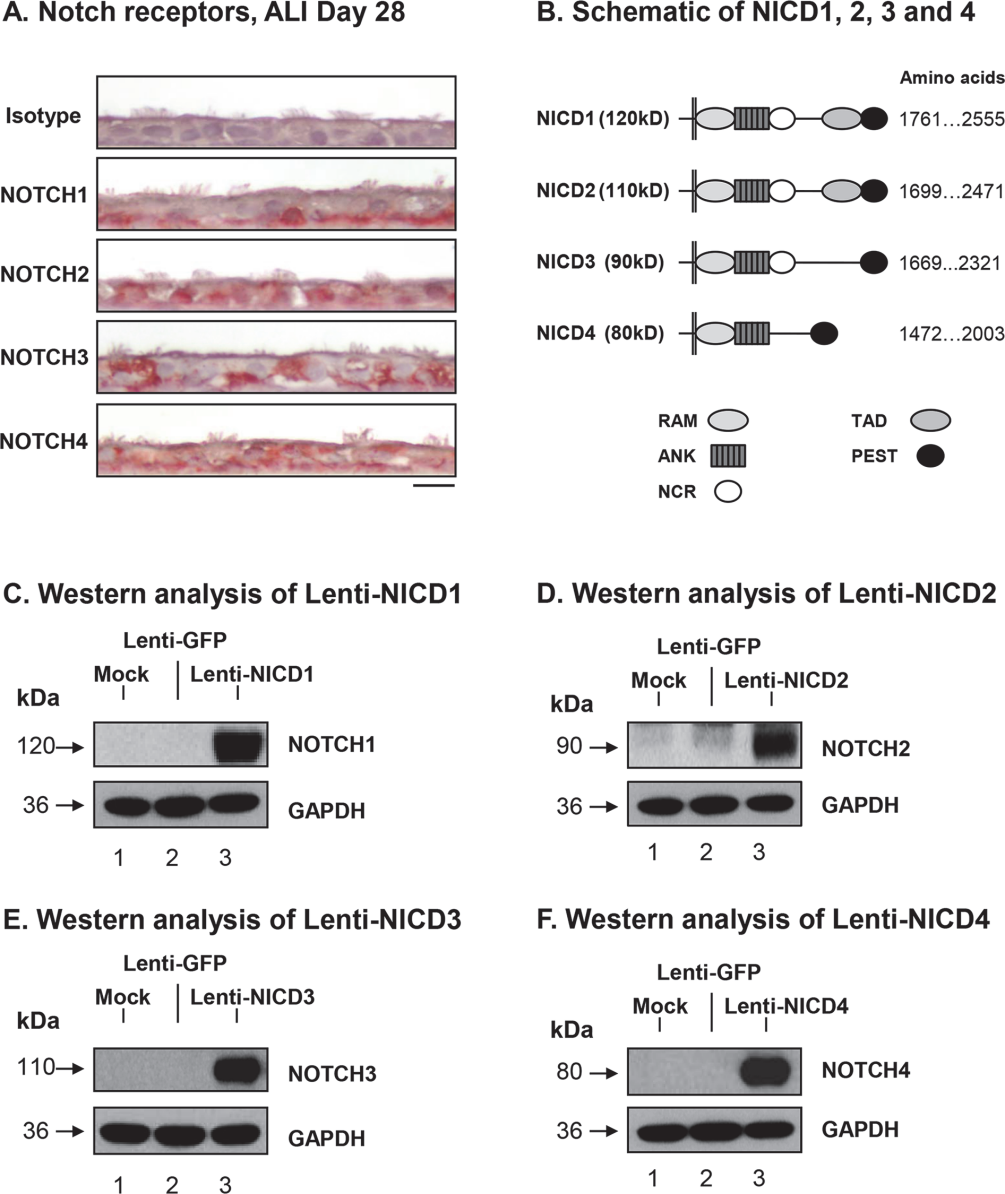
Lentivirus mediated expression of Notch intracellular domain 1–4. **A**. Localization of the Notch receptors in differentiated human airway epithelium. Immunohistochemical staining of ALI day 28 sections for NOTCH1, NOTCH2, NOTCH3 and NOTCH4. Matched isotype IgG was used as a negative control. Scale bar 20 μm. Data shown are representative images of n = 3 independent experiments. **B**. Schematic of human Notch intracellular domains (NICD) of Notch receptor 1–4. All NICDs contain a RAM23 domain (RAM), ankyrin repeats (ANK), and proline, glutamic acid, serine, threonine-rich (PEST) sequences. NICD1, 2 and 3 contain a notch cytokine response (NCR) sequence immediately C-terminal of the ankyrin repeats that regulates functional activity. Further towards the C-terminus, NICD1 and NICD2 contain C-terminal transcriptional activation domains (TAD). Amino acid sequences describe the region for each Notch receptor that encompasses the NICD. **C-F**. Western analysis of NICD1-4 from lentivirus expression constructs. Lane 1—Mock (untransfected); lane 2—Lenti-GFP; and lane 3—Lenti-NICD. Shown is the expression of **C**. NICD1, **D**. NICD2, **E**. NICD3 and **F**. NICD4. GAPDH was used as a loading control.

Activation of the Notch pathway via ligand binding to each of the four Notch receptors results in cleavage of the intracellular transmembrane region of the receptor via a γ-secretase resulting in release of the Notch intracellular domain (NICD) into the cytoplasm which subsequently translocates to the nucleus to form an active transcriptional complex [5,6]. However, to date the cell type specificity of each Notch receptor in the human airway epithelium and their role in regulating airway epithelium differentiation are unknown. Immunohistochemical staining of each Notch receptor on sections of differentiated human airway BC following 28 days of ALI culture revealed NOTCH1 expression was highly localized to the basal cell population, with weak staining in differentiated cells (Fig. 4A). Whereas, NOTCH3 was localized in a subset of basal and ciliated cells (Fig. 4A). In contrast to NOTCH1 and 3, NOTCH2 and 4 were expressed by all cell populations throughout the epithelium (Fig. 4A). To determine the effect of sustained ligand independent cell-autonomous Notch-pathway-specific signaling activation via each receptor on differentiation of human airway BC on ALI culture, sequences encompassing the intracellular domain of the human NOTCH1, 2, 3 and 4 receptors (NICD1, 2, 3 and 4, respectively) were cloned into lentiviral expression plasmids (Fig. 4B). To verify expression of each NICD, Western analysis was performed on cell lysates of 293A cells untransfected (mock) or transfected with control plasmid (Lenti-GFP) or plasmids expressing each NICD (Lenti-NICD1-4). The results confirmed expression of each NICD at the correct molecular weight (Fig. 4C-F).

**Fig 5:**
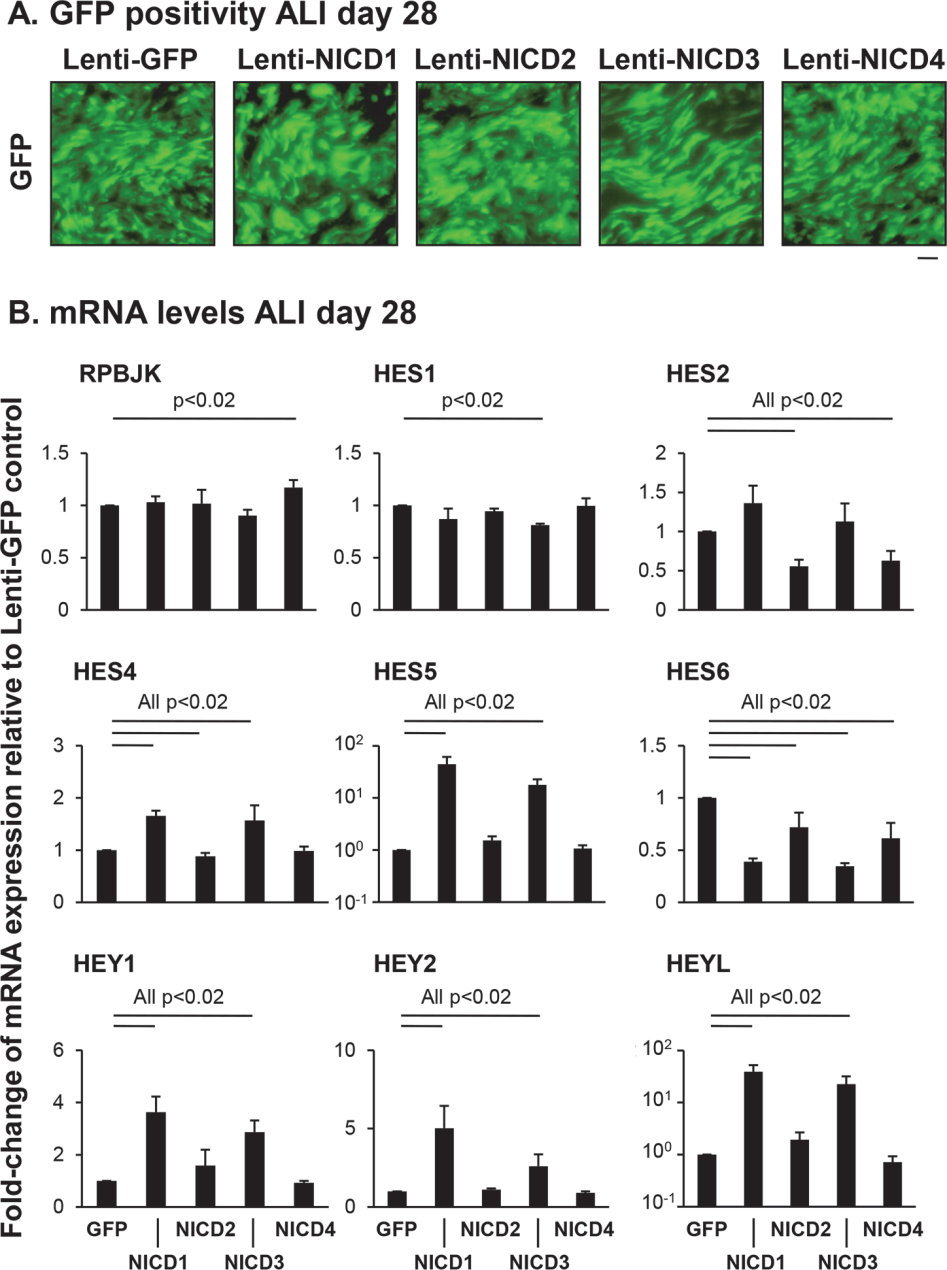
Effect of NICD-mediated sustained activation of Notch signaling on expression of the Notch downstream effectors. **A**. Efficiency of lentivirus infection on air-liquid interface (ALI) culture. Human airway basal cells were infected with lentivirus expressing GFP (Lenti-GFP) or NICD1-4 (Lenti-NICD1-4) and cultured on ALI for 28 days. Shown are representative images of infections from experiments investigating the role of NICD1-4 expression on BC differentiation. Scale bar 20 μm. **B**. Sustained activation of Notch signaling via NICD1-4 regulates expression of the Notch downstream effectors. Human airway basal cells were infected with lentivirus expressing GFP alone, NICD1, NICD2, NICD3 or NICD4 and cultured on ALI for 28 days. The mRNA expression for Notch pathway downstream effectors (RBPJK, HES1, HES2, HES4, HES5, HES6, HEY1, HEY2, and HEYL) were analyzed by TaqMan PCR. Bars indicate the mean fold-change of mRNA expression compared to Lenti-GFP infected ALI cells from n = 4 independent experiments, each performed in triplicate. Error bars indicate standard error of the mean.

Based on the knowledge that activation of Notch signaling results in multiple downstream responses, we analyzed the changes in expression of multiple known Notch downstream effectors in differentiating BC following lentivirus expression of NICD1-4. Human airway BC were infected with either control lentivirus (Lenti-GFP) or lentivirus expressing NICD1, 2, 3 or 4 (Lenti-NICD1-4) at the time of establishing the ALI cultures and equal infectivity of each virus was confirmed by GFP positivity (Fig. 5A). At ALI day 28 of culture, the cells were harvested and the expression of multiple Notch downstream effectors assessed by TaqMan analysis with specific transcript primer-probesets against each gene (Fig. 5B). Lentivirus mediated expression of NICD2 or NICD4 resulted in significant decreases (all p<0.02) in expression of the effectors HES2 and HES6, with HES4 showing a significant decrease (p<0.02) for NICD2 only and RBPJK showing a significant increase (p<0.02) in expression for NICD4 alone. However, expression of NICD1 or NICD3 resulted in a more diverse and exaggerated response with additional changes in specific downstream effector expression (Fig. 5B). Relative to Lenti-GFP infected cells, there was a significant decrease (p<0.02) in expression of HES1 in Lenti-NICD3 infected cells only, whereas for Lenti-NICD1, there was no significant change (p>0.9) in expression. In contrast, there was significant increased expression of HES4 (p<0.02), HES5 (p<0.02), HEY1 (p<0.02), HEY2 (p<0.02) and HEYL (p<0.04) in cells infected with Lenti-NICD1 or Lenti-NICD3, whereas HES6 expression was significantly decreased (p<0.02) compared to Lenti-GFP infected cells. Among the changes in expression of downstream effectors in response to NOTCH1 and 3 activation, HES5 (44.4-fold Lenti-NICD1 and 17.7-fold Lenti-NICD3) and HEYL (39.1-fold Lenti-NICD1 and 22.4-fold Lenti-NICD3) displayed the greatest fold-change increases in expression relative to controls (Lenti-GFP; Fig. 5B). Together, these data suggest that sustained activation of Notch signaling in differentiating BC via expression of NICD1-4 results in multiple downstream responses with NOTCH1 and 3 activation demonstrating the largest number of changes in expression of downstream effectors.

### Effects on Differentiation into Secretory and Ciliated Cells

**Fig 6:**
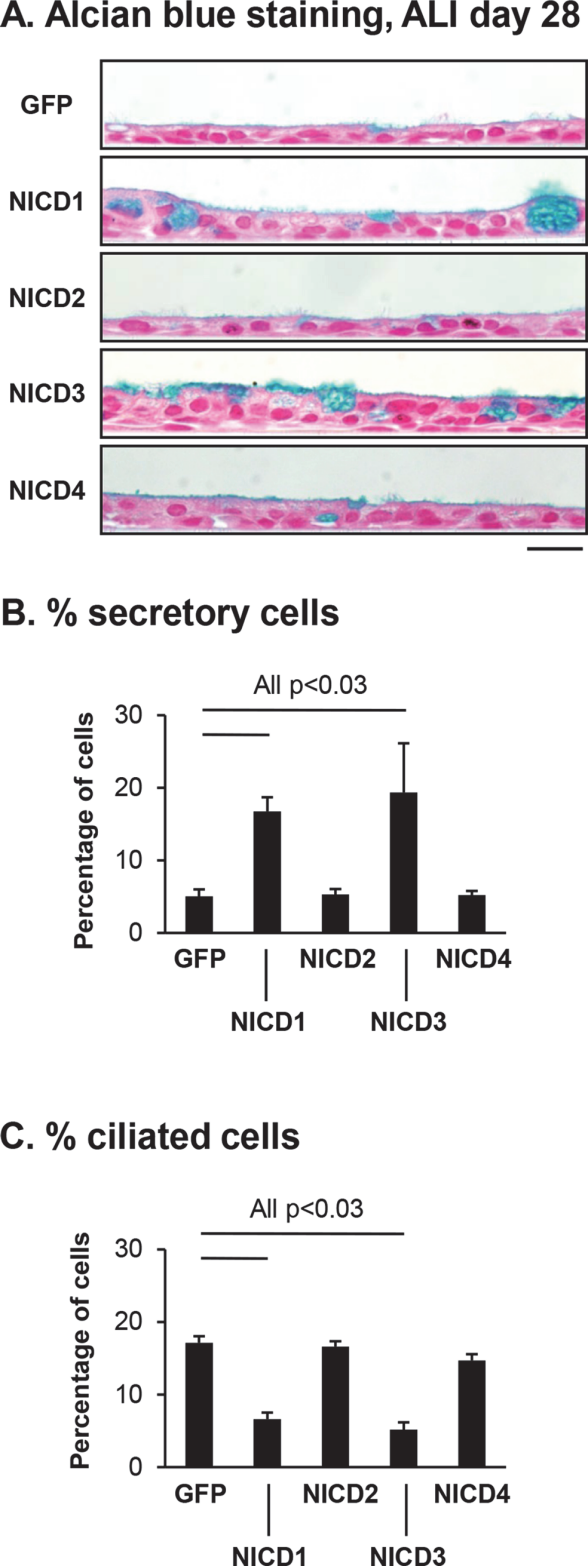
Sustained activation of Notch signaling via NICD1 or 3 skews differentiation toward the secretory lineage. **A-C**. Human airway basal cells were infected with lentivirus expressing GFP alone, NICD1, NICD2, NICD3 or NICD4 and cultured on ALI for 28 days. **A**. Alcian blue staining of ALI day 28 sections. Scale bar 20 μm. **B-C**. Quantification of secretory cells and ciliated cells on Alcian blue stained ALI day 28 sections. **B**. Percentage secretory cells; and **C**. Percentage ciliated cells. The data for **B** and **C** are the mean for n = 4 independent experiments; error bars indicate standard error of the mean.

The effect of sustained activation of each NICD on BC differentiation into a pseudostratified mucociliated epithelium was next assessed at the histological level by quantifying the number of secretory and ciliated cells by Alcian blue staining of ALI day 28 cross-sections from BC infected with either control lentivirus (Lenti-GFP) or lentivirus expressing NICD1, 2, 3 or 4 (Lenti-NICD1-4; Fig. 6A). Activation of Notch signaling with each NICD demonstrated differential functions of each receptor regarding regulating differentiation of BC into secretory and ciliated cells. Relative to control cells (Lenti-GFP), constitutive activation of Notch signaling via expression of NICD2 or NICD4 had no significant effect on regulating differentiation of BC into secretory (5.0% Lenti-GFP *vs* 5.3% Lenti-NICD2, p>0.5 and 5.0% Lenti-GFP *vs* 5.2% Lenti-NICD4, p>0.5, Fig. 6B) and ciliated cells (17.1% Lenti-GFP *vs* 16.6% Lenti-NICD2, p>0.5 and 17.1% Lenti-GFP *vs* 14.7% Lenti-NICD4, p>0.08; Fig. 6C). In contrast, constitutive activation of Notch signaling via expression of NICD1 or NICD3 resulted in a significant increase (both p<0.03) in the numbers of secretory cells relative to Lenti-GFP infected cells (5.0% Lenti-GFP *vs* 16.8% Lenti-NICD1 and 19.3% Lenti-NICD3) and a parallel significant decrease (both p<0.03) in ciliated cells (17.1% Lenti-GFP *vs* 6.6% Lenti-NICD1 and 5.2% Lenti-NICD3).

**Fig 7:**
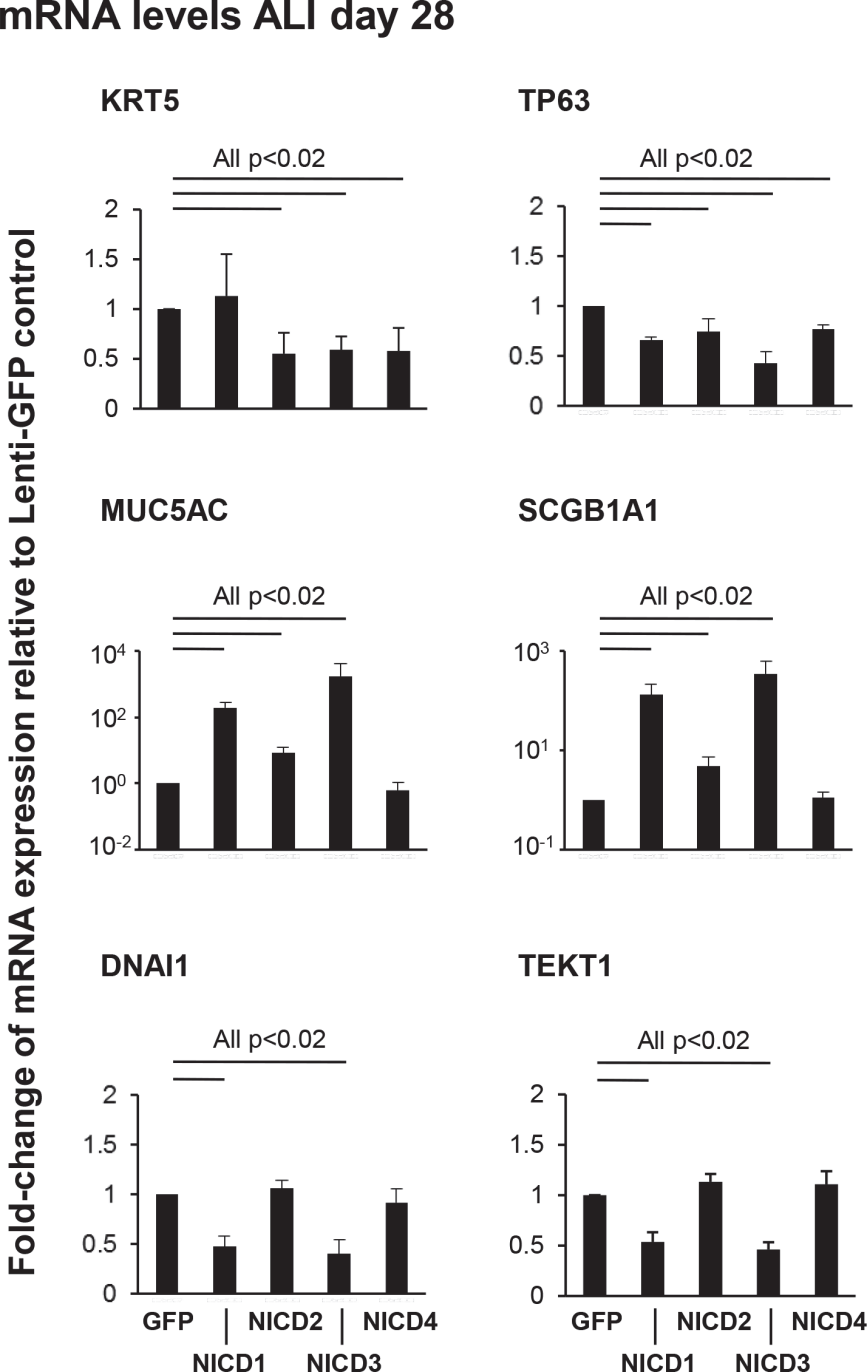
Sustained activation of Notch signaling via NICD1 or 3 skews human airway basal cell differentiation to secretory cells. Human airway basal cells were infected with lentivirus expressing GFP alone, NICD1, NICD2, NICD3 or NICD4 and cultured on ALI for 28 days and differentiation assessed by mRNA expression of cell type specific markers. TaqMan PCR was used to assess expression of basal cell markers (KRT5 and TP63); secretory cell markers (MUC5AC and SCGB1A1) and ciliated cell markers (DNAI1 and TEKT1). Bars indicate the mean fold-change of mRNA expression compared to Lenti-GFP infected ALI cells from n = 4 independent experiments, each performed in triplicate. Error bars indicate standard error of the mean.

To further characterize the effects of NICD1-4 expression on the differentiation capacity of airway BC, TaqMan quantitative PCR using primer-probesets for cell type specific markers was performed (Fig. 7). Relative to Lenti-GFP infected cells, analysis of the BC marker KRT5 revealed no significant difference in expression in cells infected with Lenti-NICD1 (p>0.2), but a significant decrease in expression in cells infected with Lenti-NICD2, NICD3 or NICD4 (all p<0.02). For the BC marker TP63, there was a significant decrease (all p<0.02) in expression in Lenti-NICD1-4 infected cells. Consistent with the histological differentiation data, analysis of the secretory cell markers MUC5AC and SCGB1A1 revealed significant increased expression (both p<0.02) in cells infected with Lenti-NICD1 or Lenti-NICD3 compared to Lenti-GFP. As expected, no significant differences (both p>0.2) in expression of MUC5AC and SCGB1A1 were observed for Lenti-NICD4. Surprisingly, a significant increase (both p<0.02) for MUC5AC (8.2-fold) and SCGB1A1 (4.8-fold) expression was observed in Lenti-NICD2 infected cells relative to Lenti-GFP. However, these fold-changes in expression observed for NICD2 were significantly lower (all p<0.05) than those observed in Lenti-NICD1 (195-fold MUC5AC and 131-fold SCGB1A1) and Lenti-NICD3 (1747-fold MUC5AC and 343-fold SCGB1A1) infected cells. Analysis of the ciliated cell markers DNAI1 and TEKT1 revealed a significant decrease (both p<0.02) in Lenti-NICD1 and Lenti-NICD3 infected cells and no significant difference (both p>0.2) in Lenti-NICD2 and Lenti-NICD4 infected cells relative to Lenti-GFP control, which is consistent with the histological differentiation data.

**Fig 8:**
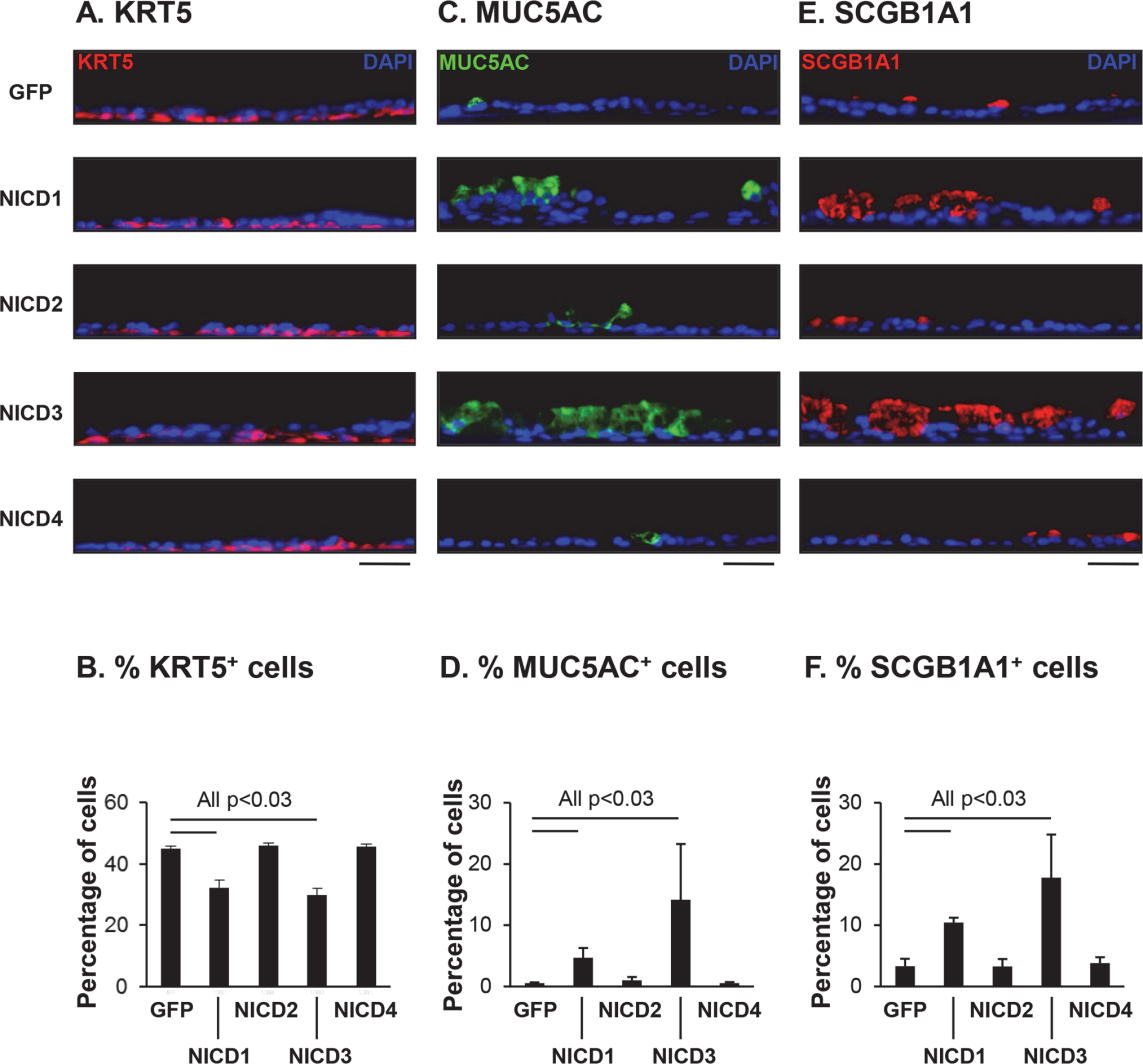
Sustained activation of Notch signaling via NICD1 or 3 increases human airway basal cell differentiation into MUC5AC and SCGB1A1 positive secretory cells. Human airway basal cells were infected with lentivirus expressing GFP alone, NICD1, NICD2, NICD3 or NICD4 and cultured on ALI for 28 days **A**. Immunofluorescence staining of KRT5 positive cells. Sections of cells on ALI day 28 membranes were stained for KRT5 (basal cell, red) and DAPI (nuclei, blue). Scale bar 20 μm. **B**. Quantification of KRT5 positive cells on ALI membranes. **C**. Immunofluorescence staining of MUC5AC positive cells. Sections of cells on ALI day 28 membranes were stained for MUC5AC (secretory cell, green) and DAPI (nuclei, blue). Scale bar 20 μm. **D**. Quantification of MUC5AC positive cells on ALI membranes. **E**. Immunofluorescence staining of SCGB1A1 positive cells. Sections of cells on ALI day 28 membranes were stained for SCGB1A1 (secretory cell, red) and DAPI (nuclei, blue). Scale bar 20 μm. **F**. Quantification of SCGB1A1 positive cells on ALI membranes. The data for **B, D** and **F** are the mean for n = 4 independent experiments; error bars indicate standard error of the mean.

Further validation of the KRT5, MUC5AC and SCGB1A1 mRNA expression data at the protein level was performed by immunofluorescent staining of each protein and quantification of cell numbers. Staining of KRT5 demonstrated a significant (all p<0.03) decrease in the number of KRT5 positive cells for both Lenti-NICD1 and Lenti-NICD3 infected cells relative to Lenti-GFP (44.7% Lenti-GFP *vs* 32.1% Lenti-NICD1 and 29.7% Lenti-NICD3). In contrast, for Lenti-NICD2 and Lenti-NICD4, there were no significant (all p>0.3) differences in the numbers of KRT5 positive cells (44.7% Lenti-GFP *vs* 45.9% Lenti-NICD2 and 44.5% Lenti-NICD4) (Fig. 8A, B). Therefore, the mRNA levels of KRT5 only correlated with the number of KRT5 positive cells for Lenti-NICD3 whereby a significant decrease was observed in both compared to Lenti-GFP infected cells. In contrast, no correlation was observed in cells infected with Lenti-NICD1, NICD2 and NICD4. These data demonstrate a similar trend to those observed with Notch inhibition with γ-secretase inhibitors whereby no positive correlation in the mRNA level and protein level of KRT5 was observed (Figs. 2–3).

Analysis of MUC5AC and SCGB1A1 staining demonstrated relative to Lenti-GFP infected cells, for both Lenti-NICD1 and Lenti-NICD3 there were significantly (all p<0.03) higher numbers of MUC5AC positive cells (0.6% Lenti-GFP *vs* 4.7% Lenti-NICD1 and 14.2% Lenti-NICD3) and SCGB1A1 positive cells (3.3% Lenti-GFP *vs* 10.4% Lenti-NICD1 and 17.7% Lenti-NICD3), thus confirming the mRNA data (Fig. 8C-F). In contrast, for Lenti-NICD2 and Lenti-NICD4, there were no significant (all p>0.75) differences in the numbers of MUC5AC positive cells (0.6% Lenti-GFP *vs* 1.0% Lenti-NICD2 and 0.6% Lenti-NICD4) and SCGB1A1 positive cells (3.3% Lenti-GFP *vs* 3.3% Lenti-NICD2 and 3.8% Lenti-NICD4, Fig. 8C-F). Therefore, even though a significant increase in mRNA expression for both MUC5AC and SCGB1A1 was observed in response to NICD2 expression, this did not translate into an increase in the numbers of MUC5AC and SCGB1A1 positive cells. Overall, these data demonstrate that NOTCH1 or 3 dependent signaling pathways regulate differentiation of BC into secretory and ciliated cells with sustained activation of each pathway skewing BC differentiation toward a secretory cell pathway.

### Analysis of Notch Downstream Effector Localization in Response to Notch Activation

**Fig 9:**
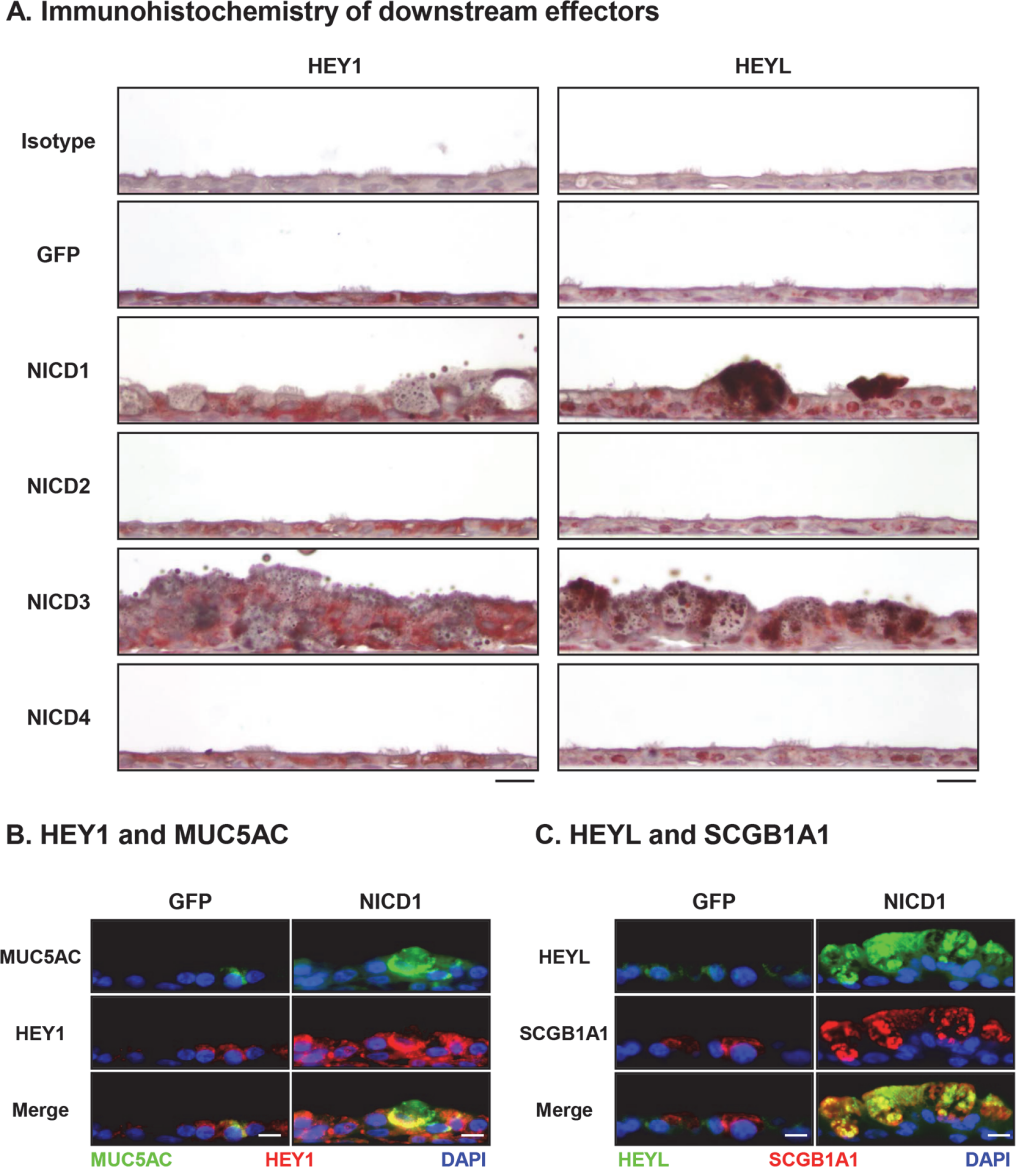
Sustained activation of Notch signaling via NICD1 or 3 increases expression of Notch downstream effectors in secretory cells. Human airway basal cells were infected with lentivirus expressing GFP alone, NICD1, NICD2, NICD3 or NICD4 and cultured on ALI for 28 days **A**. Immunohistochemical staining of HEY1 and HEYL positive cells. Sections of cells on ALI day 28 membranes were stained for HEY1 and HEYL. Matched isotype IgG was used as a negative control. Scale bar 20 μm. **B**. Immunofluorescence staining of HEY1 and MUC5AC positive cells. Sections of cells on ALI day 28 membranes were stained for MUC5AC (secretory cell, green), HEY1 (red) and DAPI (nuclei, blue). Scale bar 20 μm. **C**. Immunofluorescence staining of HEYL and SCGB1A1 positive cells. Sections of cells on ALI day 28 membranes were stained for HEYL (green), SCGB1A1 (secretory cell, red) and DAPI (nuclei, blue). Scale bar 20 μm.

To further understand the downstream effects of Notch activation in differentiating BC in response to each NICD we performed immunohistochemical analysis of ALI Day 28 cross-sections from BC infected with either control lentivirus (Lenti-GFP) or lentivirus expressing NICD1, 2, 3 or 4 (Lenti-NICD1-4) for the downstream effectors HEY1 and HEYL, both of which are upregulated at the mRNA level in response to NICD1 and NICD3 expression (Fig. 5B). The results demonstrated that HEY1 is weakly expressed in the cytoplasm of a subset of epithelium in control cells (Lenti-GFP) or cells infected with Lenti-NICD2 and NICD4 (Fig. 9A). However, in response to NICD1 and NICD3 expression there is an increase in the staining of HEY1 throughout the epithelium with elevated cytoplasmic and weak nuclear staining in secretory cells. Immunofluorescent staining of HEY1 and MUC5AC revealed increased co-localization of both proteins in cells infected with Lenti-NICD1 relative to Lenti-GFP cells (Fig. 9B). Similar to HEY1, immunohistochemical staining of HEYL demonstrated the protein is expressed weakly in the nuclei and cytoplasm of a subset of epithelium in control cells (Lenti-GFP) or cells infected with Lenti-NICD2 and NICD4 (Fig. 9A). In contrast, for cells infected with Lenti-NICD1 and NICD3 there is an increase in nuclear staining of HEYL throughout the epithelium with elevated cytoplasmic staining in secretory cells (Fig. 9A). Immunofluorescent staining of HEYL and SCGB1A1 revealed increased co-localization of both proteins in cells infected with Lenti-NICD1 relative to Lenti-GFP cells (Fig. 9C). Overall, these data demonstrate that NOTCH1 or 3 dependent signaling pathways result in up-regulation of a number of downstream effectors that co-localize with secretory cells and thus may play a role in regulating the Notch dependent skewing of BC differentiation toward a secretory cell pathway.

## Discussion

Basal cells (BC) are the proliferating stem/progenitor population that differentiate into the other specialized epithelial cell types of the airway during normal turnover and repair [26–34]. Based on the knowledge the Notch signaling pathway is expressed in the human airway epithelium [36], the present study focused on modulating the pathway to assess its role in regulating the differentiation of human airway BC. The data demonstrate the activation state of Notch signaling is essential for normal differentiation of BC into a pseudostratified mucocilated epithelium. Inhibition of Notch signaling with γ-secretase inhibitors (DAPT or DBZ) resulted in a complete block in differentiation into secretory cells, and partial block of differentiation into ciliated cells suggesting Notch activation is required for efficient luminal cell differentiation of airway BC. Constitutive activation of ligand independent cell-autonomous Notch signaling during BC differentiation via expression of the intracellular domain of each Notch receptor (NICD1-4) demonstrated only NICD1 and NICD3 altered the differentiation fate of BC into a mucociliated epithelium, with NICD2 and NICD4 having little effect. Analysis of the downstream effectors in response to NICD1-4 overexpression revealed NICD1 and 3 induced a more diverse and exaggerated effect relative to NICD2 and 4. Sustained expression of NICD1 or NICD3 skewed differentiation towards the secretory cell fate with a corresponding decrease in differentiation of ciliated cells, suggesting the levels of NOTCH1 and 3 activity are critical for determining secretory *vs* ciliated cell fate decisions. These data are consistent with previous *in vitro* studies demonstrating that activation of Notch signaling by treatment of human airway epithelial ALI cultures with DLL4 induces increased secretory cell differentiation [10]. Conversely, miRNA-mediated repression of NOTCH1 signaling increased differentiation of human airway epithelial cells into ciliated cells [38]. In support of this, a recent study demonstrated submersion of pri human bronchial epithelial cells inhibits ciliogenesis by induction of hypoxia that maintains Notch signaling which in turn represses expression of genes required for ciliogenesis [39]. Together, these data suggest that Notch signaling activity mediated by NOTCH1 and NOTCH3 receptors is essential for normal differentiation of BC into a pseudostratified mucociliated epithelium, with sustained activation skewing differentiation toward the secretory cell fate.

To date, multiple studies have demonstrated a role for Notch signaling in regulating differentiation of the murine airways into the secretory, Clara, ciliated and neuroendocrine cell types during development and in the adult lung [8–22]. For example, a recent study by Morimoto et al [15] using stepwise removal of the Notch1, 2 and 3 receptors in the airway epithelium during murine lung development demonstrated Notch2 predominantly mediates Clara/ciliated cell fate decisions with minimal contributions from Notch1 and 3. However, due to the anatomical differences between the murine and human lung, the tracheal airways of the adult mouse most closely resemble the conducting airways of the human lung, with the epithelium of both species containing basal, ciliated and secretory cells in similar ratios [32]. A study by Rock et al [13] in murine adult airway BC demonstrated that Notch signaling was active during epithelial repair and was required for differentiation of BC into luminal cells, with sustained activation via expression of NICD1 resulting in increased differentiation of MUC5AC and SCGB1A1 positive secretory cells. In support of these findings, a study by Tata et al [21] describing airway BC specific Notch activation via tetracycline inducible expression of NICD1 demonstrated increased numbers of MUC5AC positive secretory cells within 3 days of induced NICD1 expression. Our findings in the human airways are consistent with both of these murine studies. Overall, the results of our study are in agreement with those observed in BC of mice and demonstrate functional cross-species conservation of Notch signaling in regulating murine and human BC differentiation.

Due to their role as resident stem/progenitor of the airway epithelium that differentiate into the other specialized epithelial cell types of the airway during normal epithelial turnover and repair, it is likely BC play a central a role in the pathological remodeling of the airway epithelium in response to environmental stresses, including cigarette smoke [32,40]. Upon exposure to cigarette smoke, the airway epithelium becomes progressively disordered, with the decreased numbers of ciliated cells and increased numbers of mucus producing secretory cells defined as mucus cell hyperplasia [41–45]. The culmination of these changes results in enhanced mucus production and impaired mucociliary clearance leading to airflow obstruction [41,42,44]. The earliest abnormalities linked to the development of smoking induced chronic obstructive pulmonary disease (COPD) occur in the small airway epithelium [46] and the increased mucus production and accumulation in the airway of patients with COPD leads to increased rates of infection and inflammation which contribute significantly to the morbidity and mortality related with the disease [42,44,47]. *In vivo* studies have demonstrated enhanced expression of activated NOTCH1 in the airway of patients with COPD [19] and altered expression of specific Notch ligands, receptors and downstream effectors in the small airway epithelium of smokers and COPD smokers relative to healthy controls [36]. Therefore, in conjunction with our *in vitro* data we can hypothesize that cigarette smoke exposure dysregulates specific components of NOTCH1 and/or NOTCH3 mediated differentiation of the airway epithelium which may contribute to the development of a disordered airway architecture, including mucus cell hyperplasia. Thus, therapies that specifically target the NOTCH1 and 3 pathways in the airway epithelium may help restore the balance of BC differentiation to secretory and ciliated cells to maintain normal epithelial structure in smoking induced airway disorders like COPD.

## Supporting Information

S1 Fig Characterization of primary human airway basal cells. Immunohistochemical characterization of cytopreps of primary human airway basal cells isolated using selective culture methods from large airway epithelial samples obtained by bronchoscopy or purchased commercially with cell-type specific markers: KRT5 (basal cell); TP63 (basal cell); CD151 (basal cell); SCGB1A1 (secretory cell); MUC5AC (secretory cell); β-tubulin IV (ciliated cell); chromogranin A (CHGA) (neuroendocrine cell) and isotype control. Scale bar 20 μm. Data shown are representative images from a single primary donor sample obtained from each source.
(PDF)

## References

[1] Blanpain C, Horsley V, Fuchs E (2007) Epithelial stem cells: turning over new leaves. Cell 128: 445–458. 17289566 10.1016/j.cell.2007.01.014 PMC2408375

[2] Kopan R, Ilagan MX (2009) The canonical Notch signaling pathway: unfolding the activation mechanism. Cell 137: 216–233. 10.1016/j.cell.2009.03.045 19379690 PMC2827930

[3] Liu J, Sato C, Cerletti M, Wagers A (2010) Notch signaling in the regulation of stem cell self-renewal and differentiation. Curr Top Dev Biol 92: 367–409. 10.1016/S0070-2153(10)92012-7 20816402

[4] Hori K, Sen A, rtavanis-Tsakonas S (2013) Notch signaling at a glance. J Cell Sci 126: 2135–2140. 10.1242/jcs.127308 23729744 PMC3672934

[5] Andersson ER, Sandberg R, Lendahl U (2011) Notch signaling: simplicity in design, versatility in function. Development 138: 3593–3612. 10.1242/dev.063610 21828089

[6] Borggrefe T, Liefke R (2012) Fine-tuning of the intracellular canonical Notch signaling pathway. Cell Cycle 11: 264–276. 10.4161/cc.11.2.18995 22223095

[7] Iso T, Kedes L, Hamamori Y (2003) HES and HERP families: multiple effectors of the Notch signaling pathway. J Cell Physiol 194: 237–255. 12548545 10.1002/jcp.10208

[8] Ito T, Udaka N, Yazawa T, Okudela K, Hayashi H, et al (2000) Basic helix-loop-helix transcription factors regulate the neuroendocrine differentiation of fetal mouse pulmonary epithelium. Development 127: 3913–3921. 10952889 10.1242/dev.127.18.3913

[9] Shan L, Aster JC, Sklar J, Sunday ME (2007) Notch-1 regulates pulmonary neuroendocrine cell differentiation in cell lines and in transgenic mice. Am J Physiol Lung Cell Mol Physiol 292: L500–L509. 17028268 10.1152/ajplung.00052.2006

[10] Guseh JS, Bores SA, Stanger BZ, Zhou Q, Anderson WJ, et al (2009) Notch signaling promotes airway mucous metaplasia and inhibits alveolar development. Development 136: 1751–1759. 10.1242/dev.029249 19369400 PMC2673763

[11] Tsao PN, Vasconcelos M, Izvolsky KI, Qian J, Lu J, et al (2009) Notch signaling controls the balance of ciliated and secretory cell fates in developing airways. Development 136: 2297–2307. 10.1242/dev.034884 19502490 PMC2729343

[12] Morimoto M, Liu Z, Cheng HT, Winters N, Bader D, et al (2010) Canonical Notch signaling in the developing lung is required for determination of arterial smooth muscle cells and selection of Clara versus ciliated cell fate. J Cell Sci 123: 213–224. 10.1242/jcs.058669 20048339 PMC2954246

[13] Rock JR, Gao X, Xue Y, Randell SH, Kong YY, et al (2011) Notch-dependent differentiation of adult airway basal stem cells. Cell Stem Cell 8: 639–648. 10.1016/j.stem.2011.04.003 21624809 PMC3778678

[14] Tsao PN, Wei SC, Wu MF, Huang MT, Lin HY, et al (2011) Notch signaling prevents mucous metaplasia in mouse conducting airways during postnatal development. Development 138: 3533–3543. 10.1242/dev.063727 21791528 PMC3148592

[15] Morimoto M, Nishinakamura R, Saga Y, Kopan R (2012) Different assemblies of Notch receptors coordinate the distribution of the major bronchial Clara, ciliated and neuroendocrine cells. Development 139: 4365–4373. 10.1242/dev.083840 23132245 PMC3509731

[16] Xing Y, Li A, Borok Z, Li C, Minoo P (2012) NOTCH1 is required for regeneration of Clara cells during repair of airway injury. Stem Cells 30: 946–955. 10.1002/stem.1059 22331706 PMC4005608

[17] Xu K, Moghal N, Egan SE (2012) Notch signaling in lung development and disease. Adv Exp Med Biol 727: 89–98. 10.1007/978-1-4614-0899-4_7 22399341

[18] Guha A, Vasconcelos M, Cai Y, Yoneda M, Hinds A, et al (2012) Neuroepithelial body microenvironment is a niche for a distinct subset of Clara-like precursors in the developing airways. Proc Natl Acad Sci U S A 109: 12592–12597. 10.1073/pnas.1204710109 22797898 PMC3412014

[19] Boucherat O, Chakir J, Jeannotte L (2012) The loss of Hoxa5 function promotes Notch-dependent goblet cell metaplasia in lung airways. Biol Open 1: 677–691. 10.1242/bio.20121701 23213461 PMC3507293

[20] Li A, Chan B, Felix JC, Xing Y, Li M, et al (2013) Tissue-dependent consequences of Apc inactivation on proliferation and differentiation of ciliated cell progenitors via Wnt and notch signaling. PLoS One 8: e62215 10.1371/journal.pone.0062215 23646120 PMC3639955

[21] Tata PR, Pardo-Saganta A, Prabhu M, Vinarsky V, Law BM, et al (2013) Airway-specific inducible transgene expression using aerosolized doxycycline. Am J Respir Cell Mol Biol 49: 1048–1056. 10.1165/rcmb.2012-0412OC 23848320 PMC3931107

[22] Zhang S, Loch AJ, Radtke F, Egan SE, Xu K (2013) Jagged1 is the major regulator of Notch-dependent cell fate in proximal airways. Dev Dyn 242: 678–686. 10.1002/dvdy.23965 23526493

[23] Knight DA, Holgate ST (2003) The airway epithelium: structural and functional properties in health and disease. Respirology 8: 432–446. 14708552 10.1046/j.1440-1843.2003.00493.x

[24] Crystal RG, Randell SH, Engelhardt JF, Voynow J, Sunday ME (2008) Airway epithelial cells: current concepts and challenges. Proc Am Thorac Soc 5: 772–777. 10.1513/pats.200805-041HR 18757316 PMC5820806

[25] Tam A, Wadsworth S, Dorscheid D, Man SF, Sin DD (2011) The airway epithelium: more than just a structural barrier. Ther Adv Respir Dis 5: 255–273. 10.1177/1753465810396539 21372121

[26] Schoch KG, Lori A, Burns KA, Eldred T, Olsen JC, et al (2004) A subset of mouse tracheal epithelial basal cells generates large colonies in vitro. Am J Physiol Lung Cell Mol Physiol 286: L631–L642. 12959927 10.1152/ajplung.00112.2003

[27] Hong KU, Reynolds SD, Watkins S, Fuchs E, Stripp BR (2004) Basal cells are a multipotent progenitor capable of renewing the bronchial epithelium. Am J Pathol 164: 577–588. 14742263 10.1016/S0002-9440(10)63147-1 PMC1602270

[28] Hajj R, Baranek T, Le NR, Lesimple P, Puchelle E, et al (2007) Basal cells of the human adult airway surface epithelium retain transit-amplifying cell properties. Stem Cells 25: 139–148. 17008423 10.1634/stemcells.2006-0288

[29] Hackett TL, Shaheen F, Johnson A, Wadsworth S, Pechkovsky DV, et al (2008) Characterization of side population cells from human airway epithelium. Stem Cells 26: 2576–2585. 10.1634/stemcells.2008-0171 18653771 PMC2849005

[30] Rock JR, Onaitis MW, Rawlins EL, Lu Y, Clark CP, et al (2009) Basal cells as stem cells of the mouse trachea and human airway epithelium. Proc Natl Acad Sci U S A 106: 12771–12775. 10.1073/pnas.0906850106 19625615 PMC2714281

[31] Cole BB, Smith RW, Jenkins KM, Graham BB, Reynolds PR, et al (2010) Tracheal Basal cells: a facultative progenitor cell pool. Am J Pathol 177: 362–376. 10.2353/ajpath.2010.090870 20522644 PMC2893679

[32] Rock JR, Randell SH, Hogan BL (2010) Airway basal stem cells: a perspective on their roles in epithelial homeostasis and remodeling. Dis Model Mech 3: 545–556. 10.1242/dmm.006031 20699479 PMC2931533

[33] Hackett NR, Shaykhiev R, Walters MS, Wang R, Zwick RK, et al (2011) The human airway epithelial basal cell transcriptome. PLoS One 6: e18378 10.1371/journal.pone.0018378 21572528 PMC3087716

[34] Kumar PA, Hu Y, Yamamoto Y, Hoe NB, Wei TS, et al (2011) Distal airway stem cells yield alveoli in vitro and during lung regeneration following H1N1 influenza infection. Cell 147: 525–538. 10.1016/j.cell.2011.10.001 22036562 PMC4040224

[35] Paul MK, Bisht B, Darmawan DO, Chiou R, Ha VL, et al (2014) Dynamic changes in intracellular ROS levels regulate airway basal stem cell homeostasis through Nrf2-dependent Notch signaling. Cell Stem Cell 15: 199–214. 10.1016/j.stem.2014.05.009 24953182 PMC4127166

[36] Tilley AE, Harvey BG, Heguy A, Hackett NR, Wang R, et al (2009) Down-regulation of the notch pathway in human airway epithelium in association with smoking and chronic obstructive pulmonary disease. Am J Respir Crit Care Med 179: 457–466. 10.1164/rccm.200705-795OC 19106307 PMC2654975

[37] Walters MS, Gomi K, Ashbridge B, Moore MA, Arbelaez V, et al (2013) Generation of a human airway epithelium derived basal cell line with multipotent differentiation capacity. Respir Res 14: 135 10.1186/1465-9921-14-135 24298994 PMC3907041

[38] Marcet B, Chevalier B, Luxardi G, Coraux C, Zaragosi LE, et al (2011) Control of vertebrate multiciliogenesis by miR-449 through direct repression of the Delta/Notch pathway. Nat Cell Biol 13: 693–699. 10.1038/ncb2241 21602795

[39] Gerovac BJ, Valencia M, Baumlin N, Salathe M, Conner GE, et al (2014) Submersion and hypoxia inhibit ciliated cell differentiation in a notch-dependent manner. Am J Respir Cell Mol Biol 51: 516–525. 10.1165/rcmb.2013-0237OC 24754775 PMC4189480

[40] Shaykhiev R, Zuo WL, Chao I, Fukui T, Witover B, et al (2013) EGF shifts human airway basal cell fate toward a smoking-associated airway epithelial phenotype. Proc Natl Acad Sci U S A 110: 12102–12107. 10.1073/pnas.1303058110 23818594 PMC3718120

[41] Beasley MB (2010) Smoking-related Small airway disease—a review and update. Adv Anat Pathol 17: 270–276. 10.1097/PAP.0b013e3181e3bf97 20574172

[42] Kim V, Kelemen SE, buel-Haija M, Gaughan JP, Sharafkaneh A, et al (2008) Small airway mucous metaplasia and inflammation in chronic obstructive pulmonary disease. COPD 5: 329–338. 10.1080/15412550802522445 19353346

[43] Lumsden AB, McLean A, Lamb D (1984) Goblet and Clara cells of human distal airways: evidence for smoking induced changes in their numbers. Thorax 39: 844–849. 6505991 10.1136/thx.39.11.844 PMC459935

[44] Tam A, Sin DD (2012) Pathobiologic mechanisms of chronic obstructive pulmonary disease. Med Clin North Am 96: 681–698. 10.1016/j.mcna.2012.04.012 22793938

[45] Tilley AE, O'Connor TP, Hackett NR, Strulovici-Barel Y, Salit J, et al (2011) Biologic phenotyping of the human small airway epithelial response to cigarette smoking. PLoS One 6: e22798 10.1371/journal.pone.0022798 21829517 PMC3145669

[46] McDonough JE, Yuan R, Suzuki M, Seyednejad N, Elliott WM, et al (2011) Small-airway obstruction and emphysema in chronic obstructive pulmonary disease. N Engl J Med 365: 1567–1575. 10.1056/NEJMoa1106955 22029978 PMC3238466

[47] Hogg JC, Chu F, Utokaparch S, Woods R, Elliott WM, et al (2004) The nature of small-airway obstruction in chronic obstructive pulmonary disease. N Engl J Med 350: 2645–2653. 15215480 10.1056/NEJMoa032158

