# Supplementary material for: Activation of NOTCH1 or NOTCH3 Signaling Skews Human Airway Basal Cell Differentiation toward a Secretory Pathway
Source: PLoS One. 2015 Feb 20;10(2):e0116507. doi: 10.1371/journal.pone.0116507 (PMC4336283; doi:10.1371/journal.pone.0116507)
Supplement: S1 Fig — Immunohistochemical characterization of cytopreps of primary human airway basal cells isolated using selective culture methods from large airway epithelial samples obtained by bronchoscopy or purchased commercially with cell-type specific markers: KRT5 (basal cell); TP63 (basal cell); CD151 (basal cell); SCGB1A1 (secretory cell); MUC5AC (secretory cell); β-tubulin IV (ciliated cell); chromogranin A (CHGA) (neuroendocrine cell) and isotype control. Scale bar 20 μm. Data shown are representative images from a single primary donor sample obtained from each source. (PDF) [file pone.0116507.s001.pdf]

Figure S1

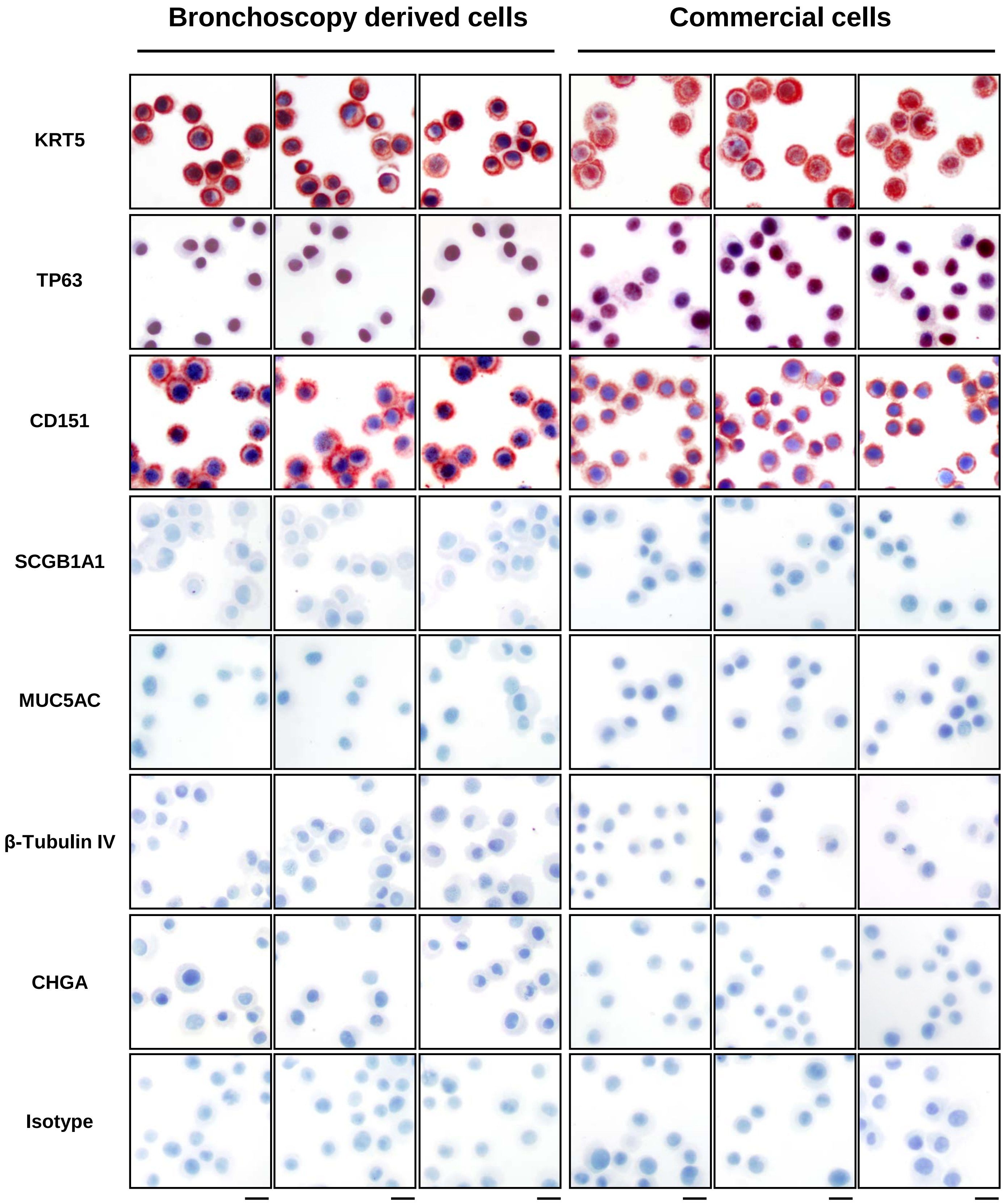
Bronchoscopy derived cells
Commercial cells
KRT5
TP63
CD151
SCGB1A1
MUC5AC
β-Tubulin IV
CHGA
Isotype
